# Exogenous NAA and IBA Alleviate the Adverse Effects of Gamma Radiation Stress and Increase Rice Seedling Survival Rates by Modulating Redox Balance and Metabolomic Regulation

**DOI:** 10.3390/antiox15101359

**Published:** 2026-10-20

**Authors:** Yanting Lu, Mengchao Zhang, Shuang Liang, Mingming Wu, Jing Ye, Shenghai Ye, Bingkui Wang

**Affiliations:** 1 Institute of Crop and Nuclear Technology Utilization, Zhejiang Academy of Agricultural Sciences, 198 Shi-Qiao Road, Hangzhou 310021, China; luyt@zaas.ac.cn (Y.L.); zhangmc@zaas.ac.cn (M.Z.); wumm@zaas.ac.cn (M.W.); yjing@zaas.ac.cn (J.Y.); 2 State Key Laboratory for Quality and Safety of Agro-Products, Zhejiang Academy of Agricultural Sciences, 198 Shi-Qiao Road, Hangzhou 310021, China; liangs@zaas.ac.cn

**Keywords:** rice seeds, radiation mutagenesis, seed treatment with auxin, redox balance, metabolic regulation

## Abstract

To investigate the effects of exogenous growth regulators on the growth of rice seedlings from irradiated seeds and to provide a reference for mutation breeding, Zhejing 100 rice seeds were irradiated with gamma rays at the median lethal dose and then soaked in solutions containing naphthalene acetic acid (NAA), indolebutyric acid (IBA), or NAA and IBA. Soaking seeds in a solution containing NAA alone or combined with IBA significantly decreased superoxide anion (O_2_^•−^), hydrogen peroxide (H_2_O_2_), and malondialdehyde contents in rice seedlings. Treatments with exogenous growth regulators at appropriate concentrations significantly enhanced antioxidant enzyme activities (e.g., superoxide dismutase, peroxidase, and ascorbate peroxidase) as well as proline, ascorbic acid, and glutathione contents, thereby increasing seedling survival rates after radiation-induced mutagenesis. Non-targeted metabolomic analyses revealed that in seedlings from irradiated seeds treated with NAA, IBA, or NAA and IBA, the two most abundant metabolite categories were lipids/lipid-like molecules and phenylpropanoids/polyketides, both of which are closely associated with antioxidant metabolism in plants. NAA and IBA also participated in the regulation of flavone and flavonol biosynthesis, flavonoid biosynthesis, purine metabolism, and zeatin biosynthesis, with positive effects on the ability of seedlings to repair damage due to irradiation. Soaking irradiated seeds in an NAA solution (0.2–0.4 mg/L) or a solution containing both NAA (0.1–0.4 mg/L) and IBA (0.25–1.0 mg/L) yielded the best results.

## 1. Introduction

Rice is a staple food for nearly half of the global population, making stable rice production critical for food security [1]. As a core rice-producing region, China maintains a stable rice production scale and is among the global leaders in terms of rice production efficiency, playing a pivotal role in the global rice industry [2]. The breeding of new varieties is crucial for rice production and development, with germplasm resources forming the foundation of rice breeding. Rice genetic improvements and breeding breakthroughs depend on the efficient development and use of rice germplasm resources. Since the mid-twentieth century, China’s rice industry has been transformed by the following three key technological innovations: Green Revolution, marked by the breeding of dwarf varieties; three-line hybrid rice technology based on cytoplasmic male sterility; and *indica*–*japonica* intersubspecific hybridization technology involving wide-compatibility genes [2,3]. The application of these technologies to analyze and exploit superior and elite germplasm resources led to the development of numerous elite varieties as well as substantial increases in rice yield and quality, thereby contributing to global food security [1,4]. Rice production worldwide is currently facing new challenges. For example, extreme weather events have resulted in frequent flooding, extreme heat, prolonged drought, and other environmental disasters, all of which are major challenges to the stress tolerance of rice [5,6]. Moreover, consumers increasingly demand high-quality rice with superior eating and cooking qualities [7]. Sustainable agricultural development calls for the promotion of rice varieties with high nitrogen-use efficiency and low carbon emissions [8,9]. Breeders also require elite germplasm resources resistant or highly tolerant to biotic and abiotic stresses, including pathogens, insect pests, drought, heat, cold, salinity, alkalinity, and heavy metal contamination, to develop new rice varieties [10,11,12,13,14,15]. Therefore, there is a continuous need for novel germplasm, with radiation mutagenesis being one of the key approaches to developing these resources [16]. The application of radiation mutagenesis over decades in China has generated mutant germplasm with desirable or specific traits related to agronomic performance, growth duration, grain quality, stress resistance, and other traits, providing a diverse material base for breeding [17,18].

Irradiating rice seeds with an appropriate dose of gamma rays can induce changes in their DNA sequence, leading to genetic mutations that can result in numerous new germplasm resources [16,18,19]. However, irradiating rice seeds can also lead to cell damage and metabolic disorders [20,21]. Generally, dry rice seeds have a moisture content of approximately 14%. In rice seeds and seedlings, radiation treatments can ionize water molecules (H_2_O^+^), which can subsequently generate numerous free radicals (e.g., O_2_^•−^ and ^•^OH). Water radiolysis-related production of secondary reactive oxygen species (ROS), such as the superoxide anion (O_2_^•−^), hydroperoxide radical (HO_2_^•^), hydroxyl radical (•OH), and hydrogen peroxide (H_2_O_2_), can result in lipid peroxidation, protein and DNA modifications, chromosomal abnormalities, and various metabolic pathway changes [22,23]. In severe cases, this can lead to the death of rice seedlings [16,24]. Therefore, inducing mutations using radiation represents an innovative approach to developing germplasm resources for breeders, but radiation is considered to be an abiotic stress for rice plants [25,26]. We previously revealed the importance of ROS production and the corresponding antioxidative processes for plant mutagenesis [18,23].

Metabolites directly contribute to plant stress responses; changes in metabolite type and abundance are regarded as the ultimate plant response to environmental stresses, including radiation. Considering its rapid development, metabolomics technology has been widely applied to qualitative and quantitative analyses of metabolites in different organisms [27]. For example, it has been used to study plant responses to abiotic and biotic stresses [28]. Additionally, Wang et al. used metabolomics technology for the metabolomic profiling of waxy corn (*Zea mays* L. *sinensis Kulesh*) leaves under high-temperature stress conditions [29]. They determined that multiple metabolic pathways, including α-linolenic acid metabolism, flavonoid biosynthesis, linoleic acid metabolism, anthocyanin biosynthesis, and arginine biosynthesis, are involved in the waxy corn response to heat stress. Akilan et al. reported that sodicity-tolerant rice uses energy relatively efficiently through the tricarboxylic acid cycle and γ-aminobutyric acid shunt, while also revealing that effective sucrose homeostasis can minimize carbon leakage from dead cells [30]. Therefore, metabolomics technology can accurately and comprehensively capture metabolite profile dynamics of plants exposed to stress. By applying it to investigate rice radiation-induced mutagenesis, it may be relevant to improving the survival rate of irradiated rice seedlings.

Both naphthalene acetic acid (NAA) and indolebutyric acid (IBA) enhance the growth-related parameters and productivity of rice and other crops [31,32]. Specifically, NAA and IBA at very low concentrations stimulate cell growth and elongation to promote plant growth and development [33]. However, whether NAA and/or IBA can alleviate stress-related damage during radiation mutagenesis-based breeding of rice has not been investigated. To the best of our knowledge, this is the first study to assess whether NAA and/or IBA can increase the rice seedling survival rate by decreasing radiation-induced damage. The study findings may provide the theoretical basis and technical insight relevant to optimizing the application of NAA and/or IBA to enhance the survival of mutagenized rice seedlings.

## 2. Materials and Methods

### 2.1. Plant Materials and Radiation Treatments

Zhejing 100 *japonica* rice seeds were obtained from the Institute of Crop and Nuclear Technology Utilization, Zhejiang Academy of Agricultural Sciences, Hangzhou, China. Each seed sample weighed 600 g. For the most uniform irradiation, seeds were divided into 12 centrifuge tubes and then thoroughly mixed. They were then irradiated at room temperature (20 ± 1 °C) using a cesium-137 (^137^Cs) source (Zhejiang Radiation Center, Hangzhou, China), with 318 Gy (i.e., LD_50_ for Zhejing 100) applied at 60 Gy/h (75 cm from the ^137^Cs source with 21,011 Ci). This dose was considered capable of inducing the maximum number of mutations while achieving a relatively high survival rate [18,23]. The absorbed dose was tracked using a ferrous sulfate (Fricke) dosimeter (320.4 ± 4.7 Gy); the error is far below the relevant standards [34].

For auxin treatments, seeds were soaked in NAA solutions at 0.02, 0.05, 0.1, 0.2, 0.4, and 0.8 mg/L (designated as A1–A6, respectively) as well as IBA solutions at 0.06, 0.15, 0.25, 0.5, 1.0, and 2.0 mg/L (designated as B1–B6, respectively). Solutions C1–C6 consisted of equal-volume mixtures of both NAA and IBA (A1 + B1, A2 + B2, A3 + B3, A4 + B4, A5 + B5, and A6 + B6). Seeds were soaked in auxin solutions (or distilled water as the control) for 48 h at a seed: solution ratio of 1:3 (*w*/*v*). Control groups were set as follows: CK1, non-irradiated seeds soaked in distilled water; CK2, irradiated seeds soaked in distilled water. After soaking, seeds were rinsed three times with distilled water and then placed in acrylonitrile–butadiene–styrene (ABS) copolymer plastic germination boxes (12 cm × 12 cm × 5 cm) lined with three layers of moistened filter paper. To ensure seeds were adequately in contact with water and uniformly absorbed water, thereby optimizing germination and seedling growth, seeds were positioned in boxes so that there was sufficient space between individual seeds. Nine boxes were prepared, each one box consisting of 100 seeds. Boxes were incubated in darkness at 28 ± 1 °C for 24 h to promote germination.

For each treatment, germinated seeds were transferred to 96-well (8 rows × 12 columns) plastic hydroponic pots (12.6 cm × 8.6 cm × 11.4 cm; Nantong Experimental Equipment Co., Ltd., Nantong, China; Figure S1). Each pot was filled with 0.9 L Yoshida nutrient solution (pH 5.8) [35,36], which was renewed every 5 days. The resulting seedlings of 96 plants per replicate were incubated in KBW400 growth chambers (Binder GmbH, Tuttlingen, Germany) set at 28 ± 1 °C, with a 12-h light (photosynthetic photon flux density of 360 μmol m^−2^ s^−1^):12-h dark cycle and 75% ± 5% relative humidity. All treatments were randomized, with three replicates per treatment. The growth status of seedlings was evaluated to determine survival rates. Moldy seeds or seedlings were removed daily.

After a 7-day germination period, the shoots of all seedlings from one ABS germination box per treatment were harvested and stored in centrifuge tubes at −86 °C until analyzed. Three replicates were used (i.e., seedlings from three separate germination boxes per treatment).

### 2.2. Seed Germination Rate and Seedling Survival Rate

Seed germination and seedling survival rates were calculated by counting healthy seedlings in plastic hydroponic pots at 7 and 28 days after sowing, respectively. Seedlings were considered healthy if they had both roots that appeared white and shoots.

### 2.3. Measurement of O_2_^•−^, H_2_O_2_, and MDA Contents

The O_2_^•−^ content was measured as described by Achary et al. and Li et al. [37,38]. Fresh shoots (0.05 g) were immersed in NBT solution, and the absorbance was recorded at 530 nm.

H_2_O_2_ levels were measured by monitoring the absorbance at 415 nm of the titanium–peroxide complex as described by Lu et al. and Lin et al. [23,39]. The Malondialdehyde (MDA) level was determined using the thiobarbituric acid reaction as described by Wu et al. [40].

### 2.4. Determination of Proline, AsA, GSH, and GSSG Contents

The proline content was determined according to the method described by Bates et al. [41]. Approximately 0.1 g fresh shoot sample was treated with 2-hydroxy-5-sulfobenzoic acid, and then the homogenate was centrifuged at 5000× *g* for 10 min. A 1-mL aliquot of the supernatant was mixed with acid ninhydrin and glacial acetic acid, after which the mixture was heated at 95 °C for 1 h. The reaction was stopped using an ice bath, and then toluene was added to the solution before measuring the absorbance at 520 nm.

Fresh shoot samples (0.1 g) were ground in 5 mL 10% (*w*/*v*) trichloroacetic acid at 0–4 °C and then centrifuged at 15,000× *g* for 10 min. The supernatant was collected and analyzed in terms of its ascorbate (AsA), GSSG, total glutathione, and glutathione (GSH) contents. Specifically, the AsA content was measured as described by Hodges et al. [42], whereas oxidized glutathione (GSSG) and total glutathione contents were measured according to the method used by Griffith [43].

### 2.5. Determination of Antioxidant Enzyme Activities

The total superoxide dismutase (SOD) activity was determined as described by Prochazkova et al. [44] and Lu et al. [18,23]. Published methods were used to determine catalase (CAT), peroxidase (POD), and ascorbate peroxidase (APX) activities [18,23].

### 2.6. Untargeted Metabolomics Profiling

To prepare extracts for metabolomic analyses, freeze-dried rice seedling shoot powder (100 mg) was mixed with 70% methanol and then sonicated on ice for 60 min. Mass spectrometry analyses were completed using a reversed-phase ACQUITY UPLC BEH C18 column (2.1 × 100 mm, 1.7 µm particle size; Waters, Milford, MA, USA) coupled to an ACQUITY I-Class UHPLC chromatography system and a VION IMS QTOF mass spectrometer equipped with an electrospray ionization interface (Waters, Milford, MA, USA). Mobile phase A (98% H_2_O, 2% acetonitrile, and 0.01% formic acid) and mobile phase B (98% acetonitrile, 2% H_2_O, and 0.01% formic acid) were used for the gradient elution. The column temperature was set at 40 °C. The flow rate was 0.4 mL/min, with an injection volume of 3 µL. Mass spectra were acquired from *m*/*z* 50 to 1000. Positive and negative electrospray ionization modes were maintained at 3 and −2.5 kV, respectively. Lock mass correction was achieved by continuously infusing a leucine–enkephalin solution at 1-min intervals while acquiring data using the MSE mode. Progenesis QI Informatics (Nonlinear Dynamics, Waters, Milford, MA, USA) was used to process data and identify compounds. Commercial databases (Metlin and NIST), plant metabolite databases, including KNApSAcK [45] and CMAUP [46] and an in-house standard library were used to identify metabolites. Compounds with a total score exceeding 30 and a fragmentation score greater than 20 were considered to be accurately annotated (Level 2). Data quality was assessed using pooled quality control (QC) samples.

### 2.7. Data Processing and Statistical Analysis

Significance was analyzed using SPSS 22.0. Differences among groups were evaluated by one-way ANOVA followed by Duncan’s test, with α = 0.05. Figures were generated using GraphPad Prism 10.3.0. Metabolomic data were normalized using probabilistic quotient normalization (PQN) and subsequently log_2_-transformed. Differentially accumulated metabolites (DAMs) were screened on the basis of *t*-tests (*p* < 0.05) and OPLS-DA models (VIP > 1 and fold change >1.5 or <0.67). Metabolic pathways were annotated using the KEGG database. For seedling survival and germination, *n* = 3 biological replicates were used per treatment. For metabolomics, *n* = 3 biological replicates were included per group with 90 plants mixed.

## 3. Results

### 3.1. Rice Seed Germination as Well as Seedling Emergence, Growth, and Morphological Characteristics

Gamma irradiation at the dosage used in this study did not significantly affect the seed germination rate (Table 1). However, it significantly altered the seedling survival rate.

Specifically, the seedling survival rate decreased from 87.3% for the non-irradiated control (CK1) to 58.3% for the irradiated control (CK2). Soaking in NAA significantly increased the seedling survival rate by 16.0–23.4%. Among the NAA treatments, A4 (0.2 mg/L) resulted in the greatest increase in the seedling survival rate; however, this increase did not differ significantly from that observed for the other NAA treatments. The IBA seed treatment significantly increased the seedling survival rate by 15.4–21.4%, with no significant differences among IBA concentrations. Soaking seeds in a solution containing both NAA and IBA significantly increased the seedling survival rate by 22.0–28.4%, which was higher than the increases resulting from the treatments with NAA or IBA alone. Significant differences were detected among the combined treatments. Specifically, the C4 treatment (0.2 mg/L NAA and 0.5 mg/L IBA) resulted in a marked increase in the seedling survival rate. Moreover, the seedling survival rate did not differ significantly between the C4 and CK1 treatments.

Soaking seeds in solutions containing different concentrations of NAA, IBA, and NAA + IBA affected rice seedling growth and morphology (Figure 1). At moderate concentrations, seedlings tended to show more vigorous growth than the untreated control (CK2), whereas at higher concentrations, seedlings appeared less vigorous. These observations were based primarily on visual assessment.

### 3.2. Lipid Peroxidation

Compared with seedlings from normal rice seeds, seedlings from irradiated seeds had significantly higher H_2_O_2_ contents (*p* ≤ 0.05) (Figure 2a–c). Treating seeds with NAA at various concentrations significantly decreased H_2_O_2_ contents (*p* ≤ 0.05). Compared with CK2, the A1–A6 treatments significantly decreased H_2_O_2_ contents by 13.25%, 23.66%, 38.12%, 33.77%, 40.09%, and 27.06%, respectively. Soaking seeds in an IBA solution also decreased H_2_O_2_ contents, with the B1–B6 treatments decreasing H_2_O_2_ contents by 9.54%, 20.88%, 34.54%, 36.34%, 25.26%, and 12.63%, respectively; these decreases were significant except for B1. A combined seed treatment with NAA and IBA significantly decreased H_2_O_2_ contents as well. Compared with CK2, the C1–C6 treatments significantly decreased H_2_O_2_ contents by 18.38%, 29.60%, 35.83%, 31.27%, 36.31%, and 21.11%, respectively.

Compared with CK1, gamma irradiation significantly increased the O_2_^•−^ contents of rice seedlings (*p* ≤ 0.05) (Figure 2d–f). Seed treatments with NAA at various concentrations significantly affected seedling O_2_^•−^ contents. Relative to the effects of CK2, the A1–A5 treatments decreased the O_2_^•−^ content by 6.68%, 14.63%, 17.78%, 17.36%, and 8.65%, respectively, with A2–A4 resulting in significant decreases (*p* ≤ 0.05). The O_2_^•−^ content after the A6 treatment was 3.03% higher than that following the CK2 treatment, but this difference was not significant. Soaking seeds in an IBA solution also decreased the O_2_^•−^ content. More specifically, the B1–B6 treatments decreased O_2_^•−^ contents by 23.01%, 26.29%, 13.82%, 11.12%, 5.62%, and 4.05%, respectively, with significant decreases observed for B1–B3 (*p* ≤ 0.05). Compared with CK2, the C1–C6 treatments decreased O_2_^•−^ contents by 9.78%, 7.70%, 11.50%, 15.70%, 22.63%, and 11.92%, respectively, with C4 and C5 resulting in significant decreases (*p* ≤ 0.05).

As an important indicator of membrane lipid peroxidation, the MDA content in seedlings from irradiated control seeds (CK2) was significantly higher than that in seedlings from non-irradiated control seeds (CK1) (*p* ≤ 0.05) (Figure 2g–i). Soaking seeds in solutions with different NAA concentrations significantly affected seedling MDA contents. Compared with CK2, the A1–A6 treatments decreased MDA contents by 7.74%, 6.50%, 15.48%, 18.89%, 20.74%, and 13.00%, respectively, with A3–A5 resulting in significant decreases (*p* ≤ 0.05). IBA seed treatments also decreased the MDA content. Compared with CK2, the B1–B6 treatments decreased MDA contents by 15.17%, 15.48%, 14.24%, 10.22%, 13.00%, and 3.10%, respectively, with significant decreases observed for B1–B3 and B5 (*p* ≤ 0.05). Soaking seeds in a solution consisting of both NAA and IBA significantly decreased MDA contents. Compared with CK2, the C1–C6 treatments decreased MDA contents by 14.35%, 11.96%, 17.39%, 22.12%, 17.96%, and 0.58%, respectively; these decreases were significant for C1–C5.

### 3.3. Antioxidant Enzyme Activities

Superoxide dismutase (SOD) is an important antioxidant enzyme. SOD activities increased significantly in rice seedlings from irradiated seeds. Compared with seedlings from CK2-treated seeds, seedlings from seeds soaked in an NAA solution had higher SOD activities to varying degrees. Specifically, the A1–A6 treatments increased SOD activities by 2.86%, 0.99%, 6.51%, 19.11%, 16.64%, and 4.04%, respectively; however, the observed increases were significant only for A4 and A5 (*p* ≤ 0.05). Soaking seeds in IBA solutions at different concentrations also increased rice seedling SOD activities, but not significantly (relative to the CK2 level). Furthermore, seed treatments with a solution containing both NAA and IBA enhanced SOD activities. Compared with CK2, the C1–C6 treatments increased SOD activities by 3.51%, 1.60%, 17.15%, 22.10%, 19.05%, and 3.54%, respectively, with C3–C5 inducing significant increases (Figure 3a–c).

Gamma irradiation also increased peroxidase (POD) activities. Compared with CK2, NAA seed treatments enhanced POD activities in rice seedlings. Specifically, the A1–A6 treatments increased POD activities by 0.52%, 4.71%, 11.51%, 13.75%, 16.87%, and 6.53%, respectively, but only A5 resulted in a significant increase (*p* ≤ 0.05). The effects of IBA solutions were similar to those of NAA solutions. POD activities following the B1–B6 treatments increased by 2.24%, 6.70%, 12.25%, 19.37%, 10.24%, and 8.82%, respectively (relative to the CK2 level), with the B4 treatment resulting in a significant increase. Soaking seeds in a solution with both NAA and IBA also enhanced seedling POD activities. Compared with CK2, the C1–C6 treatments increased POD activities by 6.37%, 13.48%, 21.41%, 21.52%, 22.31%, and 15.69%, respectively; significant increases were observed for C2–C6 (Figure 3d–f).

Gamma irradiation also promoted catalase (CAT) activities (Figure 3g–i). However, compared with CK2, NAA seed treatments decreased rice seedling CAT activities (decreased by 5.93%, 10.13%, 20.41%, 15.39%, 13.05%, and 6.12% following the A1–A6 treatments, respectively). However, these decreases were significant only for A3 and A4 (*p* ≤ 0.05). Similar to NAA seed treatments, IBA seed treatments decreased CAT activities, with B4 resulting in a significant decrease (relative to the CK2 level). In terms of the effects of a seed treatment involving a solution comprising both NAA and IBA, the C1–C6 treatments decreased CAT activities by 0.43%, 2.92%, 13.53%, 18.40%, 20.27%, and 3.11%, respectively (relative to the CK2 level), with significant decreases detected for C4 and C5.

### 3.4. Ascorbate Peroxidase (APX) Activity and Antioxidant Contents

APX is an enzyme whose activity is closely associated with ascorbic acid (AsA) and glutathione (GSH) levels. Irradiating seeds with gamma rays significantly increased rice seedling APX activities (Figure 4a–c). Compared with CK2, NAA seed treatments enhanced APX activities to varying degrees. More specifically, after the A1–A6 treatments, APX activities increased by 4.87%, 7.96%, 10.14%, 17.66%, 18.99%, and 13.52%, respectively; significant increases were observed for A4 and A5 (*p* ≤ 0.05) (Figure 4a). Soaking seeds in solutions with different IBA concentrations also improved APX activities in rice seedlings; however, the effects of IBA treatments did not differ significantly from the effect of the CK2 treatment (Figure 4b). Furthermore, a combined seed treatment with NAA and IBA enhanced APX activities. Compared with CK2, the C1–C6 treatments increased APX activities by 1.32%, 4.56%, 9.97%, 17.11%, 24.90%, and 14.52%, respectively, but significant increases were detected only for C4–C6 (*p* ≤ 0.05) (Figure 4c).

AsA is an important antioxidant. Irradiating rice seeds with gamma rays significantly decreased AsA contents in rice seedlings. Soaking seeds in NAA solutions at various concentrations increased seedling AsA contents to varying degrees (Figure 4d). Compared with CK2, the A1–A6 treatments increased AsA contents by 0.16%, 2.82%, 6.77%, 18.22%, 26.71%, and 27.73%, respectively, with A5 and A6 resulting in significant increases. IBA solutions used for seed treatments also increased seedling AsA contents (Figure 4e). The B1–B6 treatments increased AsA contents by 5.27%, 5.94%, 11.91%, 20.03%, 34.77%, and 21.39%, respectively, with the B4–B6 treatments having significant effects. Soaking seeds in a solution consisting of both NAA and IBA also increased seedling AsA contents. Compared with CK2, the C1–C6 treatments increased AsA contents by 10.05%, 19.44%, 45.38%, 35.66%, 42.79%, and 26.98%, respectively. The increases following the C2–C6 treatments were significant (*p* ≤ 0.05) (Figure 4f).

GSH is an essential component of the AsA–GSH cycle. Gamma irradiation of rice seeds significantly decreased GSH contents in rice seedlings. Soaking seeds in NAA, IBA, or NAA + IBA solutions increased seedling GSH contents; however, none of these increases were significant (relative to the CK2 level) except for A5 (Figure 4g–i). Oxidized glutathione (GSSG) also plays an important role in the AsA–GSH cycle. Compared with CK1, gamma irradiation significantly decreased seedling GSSG contents. Following seed treatments with NAA, GSSG contents in seedlings increased to varying degrees (Figure 4j). Compared with CK2, the A1–A6 treatments increased GSSG contents by 6.64%, 8.09%, 9.32%, 11.08%, 16.72%, and 17.91%, respectively, with the increases induced by A5 and A6 revealed to be significant (*p* ≤ 0.05). Soaking seeds in an IBA solution also increased GSSG contents in seedlings, but not significantly. Seed treatments with a solution containing both NAA and IBA similarly increased seedling GSSG contents (Figure 4l). Compared with CK2, the C1–C6 treatments increased GSSG contents by 4.31%, 2.96%, 9.47%, 13.33%, 19.18%, and 24.71%, respectively, with significant increases detected for C5–C6 (*p* ≤ 0.05).

Proline is a key antioxidant. Analyses of seedlings from gamma-irradiated seeds revealed significant increases in proline content (Figure 5). In terms of the effects of NAA, proline contents in seedlings from seeds that underwent the A1–A6 treatments increased by 4.91%, 9.43%, 14.84%, 23.51%, 26.47%, and 23.09%, respectively. These increases were significant for A3–A6 (relative to the CK2 level) (*p* ≤ 0.05). Soaking seeds in an IBA solution also promoted proline accumulation in the resulting seedlings; however, only B5 had a significant effect (14.9% higher than the CK2 level). Furthermore, soaking seeds in a solution that combined NAA and IBA increased proline contents in seedlings. Compared with CK2, the C1–C6 treatments increased proline contents by 2.51%, 3.75%, 8.16%, 16.09%, 17.86%, and 12.18%, respectively, with significant increases observed for C4 and C5 (*p* ≤ 0.05).

### 3.5. Metabolic Changes

After irradiating seeds, untargeted metabolomic analyses of the resulting rice seedlings revealed 689 metabolites that were annotated. The most abundant metabolites were classified as lipids and lipid-like molecules or phenylpropanoids and polyketides (Figure 6). A total of 236 differentially accumulated metabolites (DAMs) were identified between CK2 and CK1 (141 upregulated and 95 downregulated). Six metabolic pathways were significantly enriched among these DAMs (Figure 7a). A comparison between Group A and CK2 detected 56 DAMs (38 upregulated and 18 downregulated), with only one enriched metabolic pathway among these metabolites (Figure 7b). By contrast, a comparison between Group B and CK2 revealed 68 DAMs (39 upregulated and 29 downregulated), with three enriched metabolic pathways among these metabolites (Figure 7c). Finally, 59 DAMs were detected between Group C and CK2 (41 upregulated and 18 downregulated); three metabolic pathways were enriched among these metabolites (Figure 7d).

The following four significantly enriched metabolic pathways related to growth regulators were selected for the subsequent analysis: flavone and flavonol biosynthesis, flavonoid biosynthesis, zeatin biosynthesis, and purine metabolism. These pathways were associated with 14 metabolites (Figure 8). A comparison with CK1 samples revealed six upregulated and six downregulated DAMs in CK2 samples. DAMs in seedlings from irradiated seeds treated with different concentrations of NAA, IBA, or NAA + IBA recovered to varying degrees (relative to the corresponding CK2 level), indicating that exogenous auxin can alleviate the metabolic imbalance caused by radiation-induced stress.

Principal component analyses (Figure 9) revealed the clear separation of six groups along the PC1 and PC2 axes, reflecting distinct metabolic responses. For the NAA treatments, PC1 and PC2 collectively explained 80.3% of the total variance (Figure 9a). Along the PC2 axis, the metabolite accumulation profiles of A4, A5, and A6 shifted toward those of the non-irradiated control (CK1). Along the PC1 axis, the profiles of A2 and A3 also trended toward CK1 profiles. This indicates that exogenous NAA treatments had a regulatory effect on plant metabolism. For the IBA treatments, PC1 explained more of the metabolic variance (67.8%) than PC2 (21.2%) (Figure 9b). CK1 samples were distributed in the high-value region of PC2, exhibiting distinct metabolic profiles compared to all other groups. In contrast, the IBA-treated groups (B1–B6) and the irradiated control (CK2) clustered closely together, with only slight dispersion observed for B2. This proximity suggests that exogenous IBA treatment did not induce a global metabolic reprogramming distinct from the irradiated state. However, this overall similarity does not preclude specific, localized metabolic adjustments that may underlie the observed improvement in seedling survival. For the combined treatments with both NAA and IBA, PC1 and PC2 explained 57.7% and 25.3% of the metabolic variance, respectively (Figure 9c). Four groups (CK2, C1, C2, and C3) were clustered near the origin on the left side of the PC1 axis, indicating high metabolic similarity. By contrast, C4, C5, and C6 had distinct metabolite profiles along both axes (PC1 and PC2), trending toward the CK1 profile.

## 4. Discussion

Irradiating crop seeds with gamma rays at the median lethal dose (LD_50_) is the most commonly used method for radiation-based mutation breeding. According to earlier Pearson’s correlation analyses, approximately 60% of irradiated seedlings (at LD_50_) are expected to survive [18,47]. Under the experimental conditions of the current study, when irradiated seeds were not treated with growth regulators, the final seedling survival rate was 58.3% (Table 1). The key direct effect of gamma irradiation on rice seeds and seedlings is a substantial increase in ROS generation because of the interaction between gamma rays and water molecules within seeds [18,23,25]. NAA and IBA, which are two important plant hormones, regulate ROS homeostasis under stress conditions, thereby enhancing plant stress tolerance [48,49]. In the present study, we determined that treatments with NAA, IBA, or NAA and IBA were associated with significantly lowerH_2_O_2_ levels in seedlings, with the A1–A6, B2–B6, and C1–C6 treatments having particularly pronounced effects. O_2_^•−^ contents decreased significantly following the A2–A4, B1–B3, C4, and C5 treatments (relative to the CK2 level). In addition, seedling MDA contents were significantly lower in samples that underwent the A3–A5, B1–B3, B5, and C1–C5 treatments than in samples in the CK2 treatment group. In principle, the effects of auxin on these three indicators should be highly consistent, but this was in contrast to the relative inconsistency observed in this study. Although this inconsistency may be attributed to measurement errors, the internal mechanisms underlying the radiation-induced damage in rice seedlings differ from those associated with the detrimental effects of other abiotic stresses. Theoretically, crop seedling damage due to other abiotic stresses, such as drought, low temperatures, and heavy metals, exhibits a systematic consistency. By contrast, although radiation-induced abiotic stress produces consistent phenotypes at a particular dosage, the internal damage to each seedling (e.g., changes in chromosomes and base sequences as well as in organelles) is highly heterogeneous [50,51]. This heterogeneity may partly explain the observed inconsistencies in the measurements of these three indicators.

SOD, POD, and CAT are crucial antioxidant enzymes responsible for alleviating ROS-related stress [26,48]. Following gamma irradiation, SOD, POD, and CAT activities were significantly higher in rice seedlings from CK2-treated seeds than in seedlings from CK1-treated seeds. This pattern is consistent with the possibility that an increase in ROS production induced by gamma irradiation, which can activate plant defense mechanisms [25,50]. Treating seeds with NAA significantly increased seedling SOD activities, with the A4 and A5 treatments resulting in the most notable increases. By contrast, soaking seeds in an IBA solution had no significant effect on SOD activities. However, soaking seeds in a mixture of both auxins significantly enhanced SOD activities, especially following the C3–C5 treatments. Seed treatments with NAA or IBA alone or both auxins combined had similar effects on seedling POD and SOD activities; however, the A5, B4, C2–C6 treatment had a significant effect. Overall, the effects of these two auxins on SOD and POD activities were in accordance with their effects on H_2_O_2_, O_2_^•−^, and MDA contents. This is consistent with the findings of recent studies on various abiotic stresses [12,15]. Soaking seeds in NAA, IBA, or NAA + IBA solutions tended to decrease CAT activities in seedlings. Earlier research on the effects of growth regulators on CAT activities in plants under stress conditions produced conflicting results. Although some studies reported that certain growth regulators, such as NAA and IBA, enhance CAT activities, other studies had the opposite findings. The results of the current study are consistent with our previous findings regarding the effects of brassinosteroids on CAT activities in rice seedlings [18]. Moreover, they are also supported by the findings of other studies [52,53].

APX activities are highly correlated with AsA and GSH levels. The effects of gamma irradiation on APX activities, as well as the effects of NAA and IBA applied alone or together on APX activities in seedlings, were similar to the effects of these treatments on SOD and POD activities; these results were consistent with the findings of earlier research on the effects of growth regulators on APX activities in plants under stress conditions [25]. The observed changes in AsA and proline contents in seedlings from seeds soaked in NAA, IBA, and NAA + IBA solutions were in accordance with the changes in H_2_O_2_, O_2_^•−^, and MDA contents; similar results were obtained in a previous study [54]. Although the overall differences in GSH and GSSG contents in seedlings derived from irradiated seeds soaked in one of three auxin solutions were not significant, the observed trends were consistent with those of AsA contents. Moreover, they were similar to the findings of previous related studies [23,25,54].

During plant responses to stresses, including radiation, metabolic networks are reprogrammed, resulting in the differential accumulation of specific metabolites. Therefore, elucidating metabolite profiles under stress conditions has become a key approach to unraveling the molecular mechanisms mediating plant stress tolerance [55]. In the present study, the metabolomes of irradiated samples treated with NAA, IBA, and NAA + IBA were analyzed. The two most abundant metabolite categories were lipids/lipid-like molecules and phenylpropanoids/polyketides (Figure 6). Both metabolite classes are closely associated with plant antioxidant metabolism [56]. Previous studies have reported that flavonoids and purines play a pivotal role in mitigating stress-associated chemical changes caused by biotic and abiotic factors, including gamma radiation, thereby providing stress protection [57,58]. Notably, flavonoids exhibit antioxidative activity by detoxifying ROS produced under stress conditions. These reports are consistent with and support the findings of the present study. Further comparative analyses of CK1 and CK2 treatment groups revealed 236 DAMs that were significantly associated with six metabolic pathways (Figure 7a). Notably, all six pathways are closely related to antioxidant metabolism, which is consistent with our previous findings [18,23] as well as the results of the present study. Analyses of DAMs in seedlings derived from irradiated seeds soaked in NAA, IBA, and NAA + IBA solutions identified the following four significantly enriched growth regulator-related metabolic pathways: flavone and flavonol biosynthesis, flavonoid biosynthesis, purine metabolism, and zeatin biosynthesis. Among these pathways, the first three are closely associated with antioxidant metabolism. The observed changes in zeatin biosynthesis may be attributed to the regulatory effects of NAA and IBA alone or together on cell division, growth, and development as well as senescence and damage repair in seedlings. However, this possibility remains to be experimentally verified. Moreover, the metabolites involved in the first three pathways have rarely been investigated in terms of their changes during the gamma irradiation-based mutagenesis of rice. Therefore, in-depth and systematic studies should be conducted to address these knowledge gaps.

Further analyses of DAMs showed that following the NAA seed treatment, the metabolite accumulation profiles of seedlings from seeds that underwent the A4–A6 treatments tended to shift toward the profiles of normal seedlings (i.e., those from CK1-treated seeds). Similar profiles were not detected for seedlings from IBA-treated seeds. In terms of the effects of the NAA + IBA seed treatment, the metabolite accumulation profiles of seedlings from seeds that underwent the C4–C6 treatments also tended to shift toward those of seedlings from CK1-treated seeds. These findings are generally consistent with the aforementioned antioxidant enzyme activities following NAA, IBA, and NAA + IBA seed treatments. However, relevant reports remain scarce, and the 7-day metabolomics data and 28-day survival endpoint are temporally mismatched, thereby necessitating additional in-depth investigations.

On the basis of a comprehensive analysis of peroxide contents, antioxidant enzyme activities, antioxidant contents, and untargeted metabolomic profiles along with the final seedling survival rate, we concluded that soaking seeds in an NAA (0.2–0.4 mg/L) solution (A4 and A5) or an NAA (0.1–0.4 mg/L) + IBA (0.25–1.0 mg/L) solution (C3, C4, and C5) can effectively mitigate gamma irradiation-induced damage, thereby enabling breeders to increase the number of surviving mutant rice seedlings.

## 5. Conclusions

Soaking rice seeds irradiated with gamma rays at 318 Gy (i.e., LD_50_) in a solution containing NAA at appropriate concentrations or solutions comprising both NAA and IBA effectively alleviates ROS-induced stress in seedlings. This protective effect is associated with elevated activities of antioxidant enzymes, such as SOD, POD, and APX, and increased levels of antioxidants, including proline, AsA, and GSH. Furthermore, NAA and IBA help regulate flavone and flavonol biosynthesis, flavonoid biosynthesis, purine metabolism, and zeatin biosynthesis, thereby improving the ability of seedlings to repair radiation-induced damage. Of the treatments included in this study, soaking seeds in a solution containing NAA at 0.2–0.4 mg/L or a solution consisting of NAA (0.1–0.4 mg/L) and IBA (0.25–1.0 mg/L) had the most favorable effects.

## Figures and Tables

**Figure 1:**
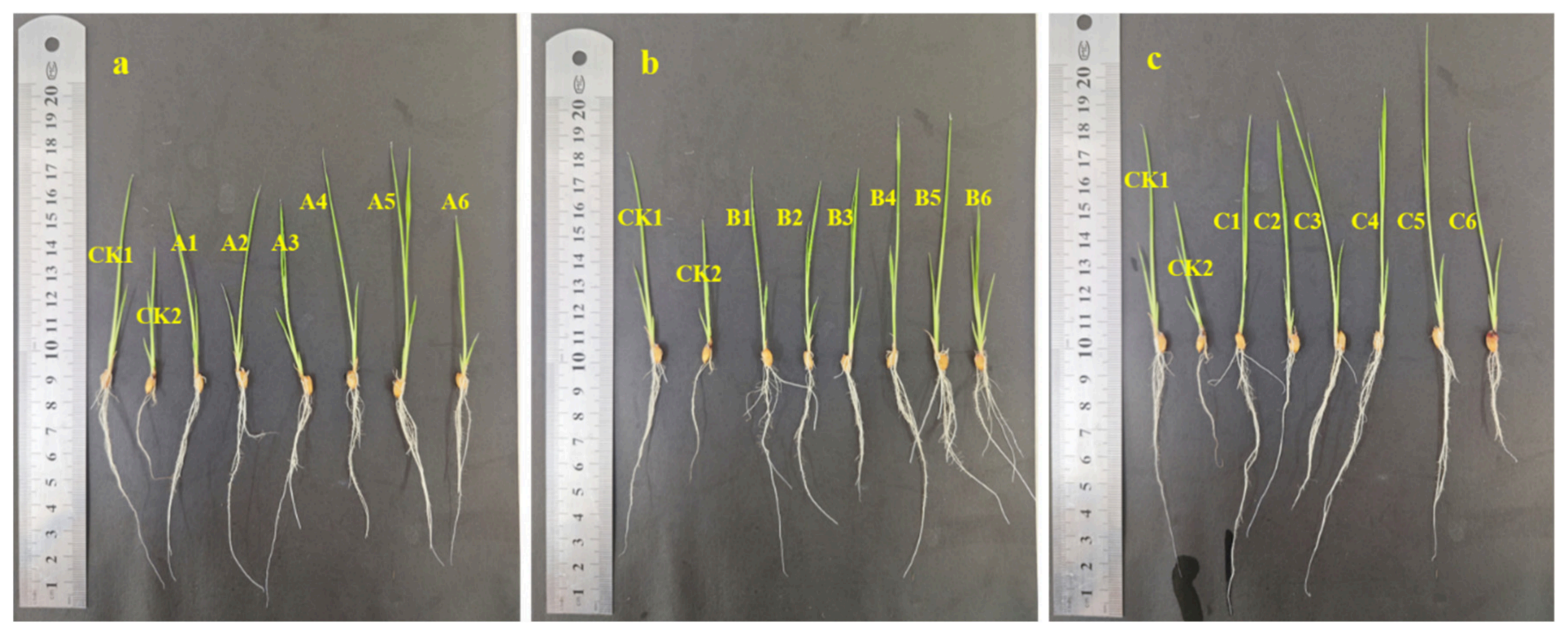
Growth and morphology of rice seedlings derived from irradiated seeds soaked in auxin solutions. (**a**): Seeds soaked in an NAA solution. From left to right: CK samples and samples treated with 0.02 (A1), 0.05 (A2), 0.1 (A3), 0.2 (A4), 0.4 (A5), and 0.8 (A6) mg/L NAA. (**b**): Seeds soaked in an IBA solution. From left to right: CK samples and samples treated with 0.06 (B1), 0.15 (B2), 0.25 (B3), 0.5 (B4), 1.0 (B5), and 2.0 (B6) mg/L IBA. (**c**): Seeds soaked in an NAA + IBA solution. From left to right: CK samples and samples treated with C1 (A1 + B1), C2 (A2 + B2), C3 (A3 + B3), C4 (A4 + B4), C5 (A5 + B5), and C6 (A6 + B6).

**Figure 2:**
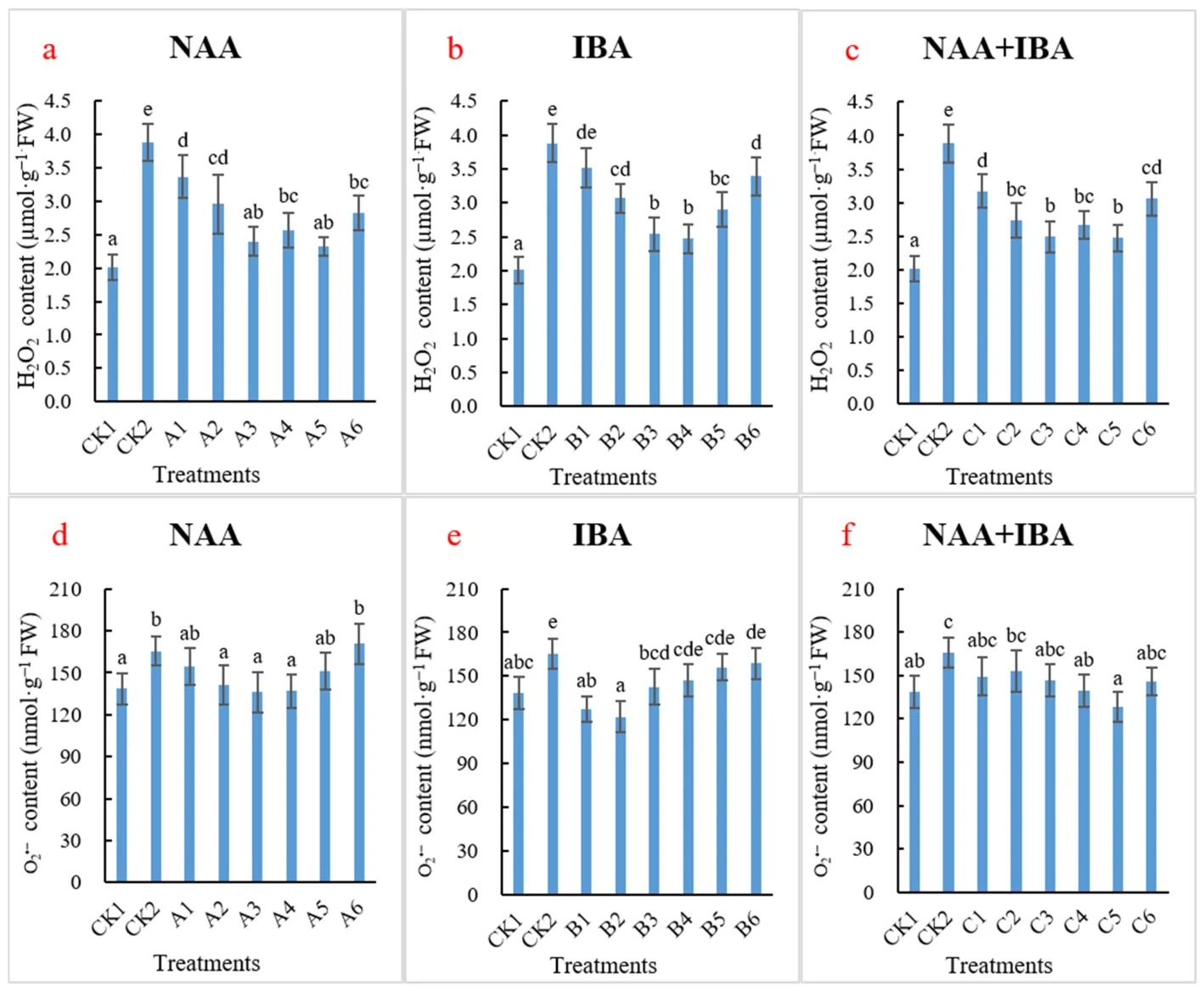

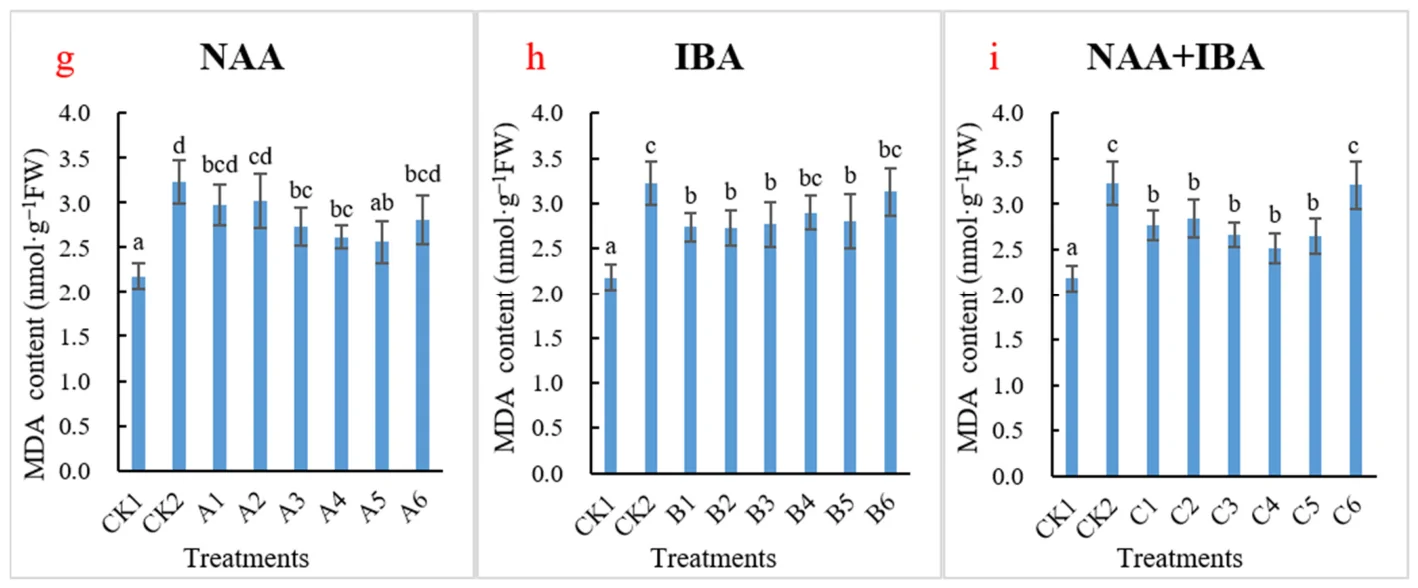
H_2_O_2_, O_2_^•−^, and MDA contents in rice seedlings derived from irradiated seeds soaked in auxin solutions. Bars with different letters indicate significant differences among treatments at the 0.05 level (Duncan), the same as in the following figures. (**a**,**d**,**g**): Seeds soaked in NAA solution at concentrations of 0.02 (A1), 0.05 (A2), 0.1 (A3), 0.2 (A4), 0.4 (A5), and 0.8 (A6) mg/L. (**b**,**e**,**h**): Seeds soaked in IBA solution at concentrations of 0.06 (B1), 0.15 (B2), 0.25 (B3), 0.5 (B4), 1.0 (B5), and 2.0 (B6) mg/L. (**c**,**f**,**i**): Seeds soaked in NAA + IBA solution at combined concentrations of C1 (A1 + B1), C2 (A2 + B2), C3 (A3 + B3), C4 (A4 + B4), C5 (A5 + B5), and C6 (A6 + B6) mg/L. CK1: normal seeds; CK2: irradiated seeds without auxin treatment.

**Figure 3:**
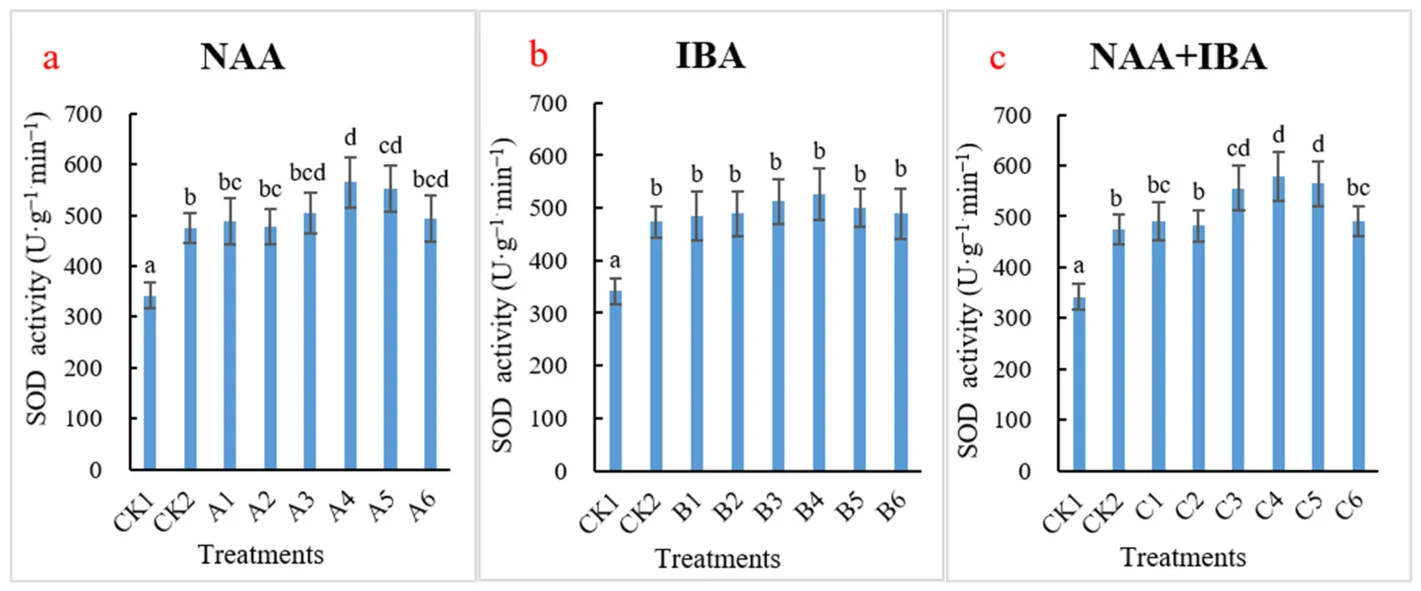

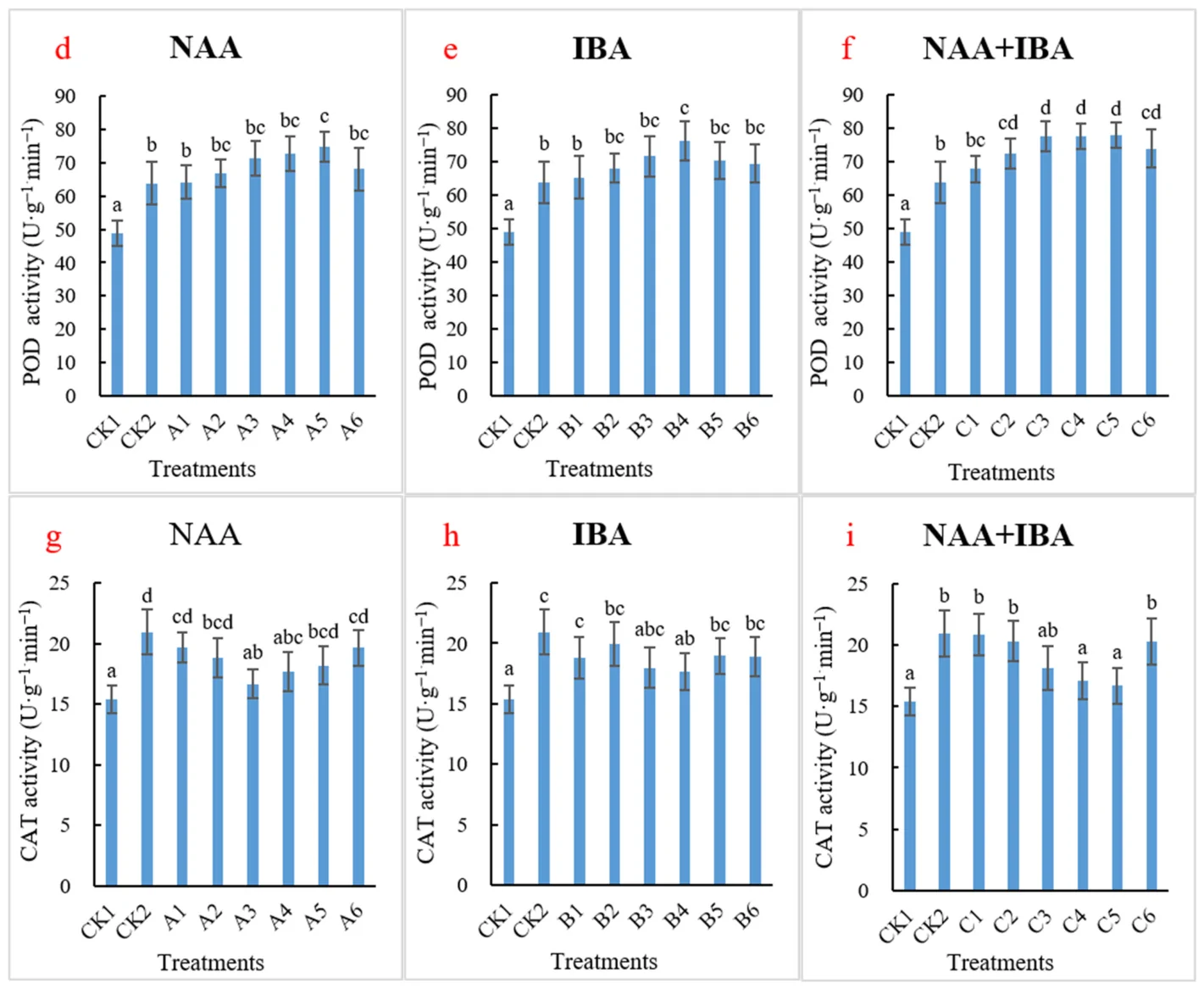
SOD, POD, and CAT activities in rice seedlings derived from irradiated seeds soaked in auxin solutions. (**a**,**d**,**g**): Seeds soaked in NAA solution at concentrations of 0.02 (A1), 0.05 (A2), 0.1 (A3), 0.2 (A4), 0.4 (A5), and 0.8 (A6) mg/L. (**b**,**e**,**h**): Seeds soaked in IBA solution at concentrations of 0.06 (B1), 0.15 (B2), 0.25 (B3), 0.5 (B4), 1.0 (B5), and 2.0 (B6) mg/L. (**c**,**f**,**i**): Seeds soaked in NAA + IBA solution at combined concentrations of C1 (A1 + B1), C2 (A2 + B2), C3 (A3 + B3), C4 (A4 + B4), C5 (A5 + B5), and C6 (A6 + B6) mg/L. CK1: normal seeds; CK2: irradiated seeds without auxin treatment.

**Figure 4:**
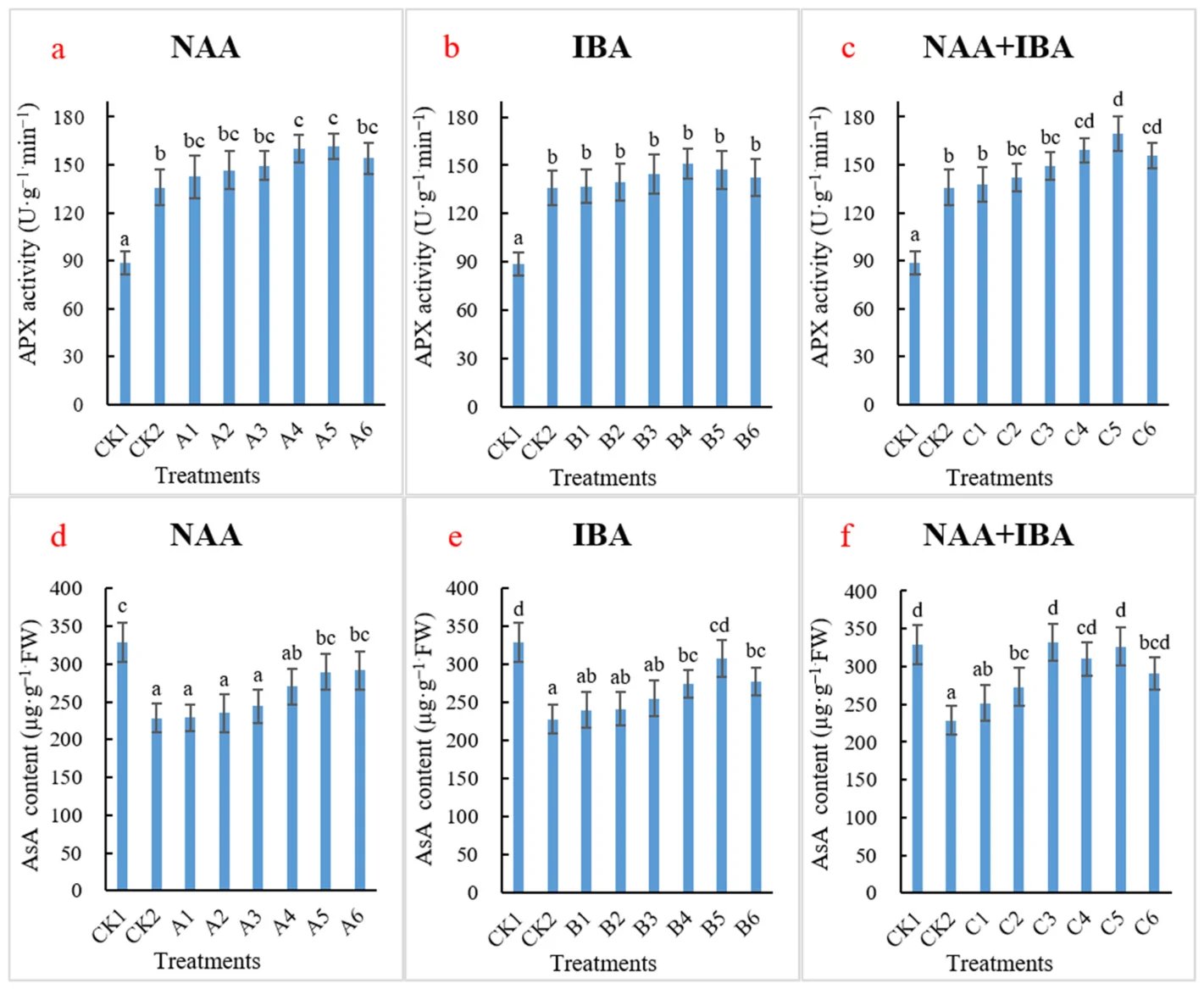

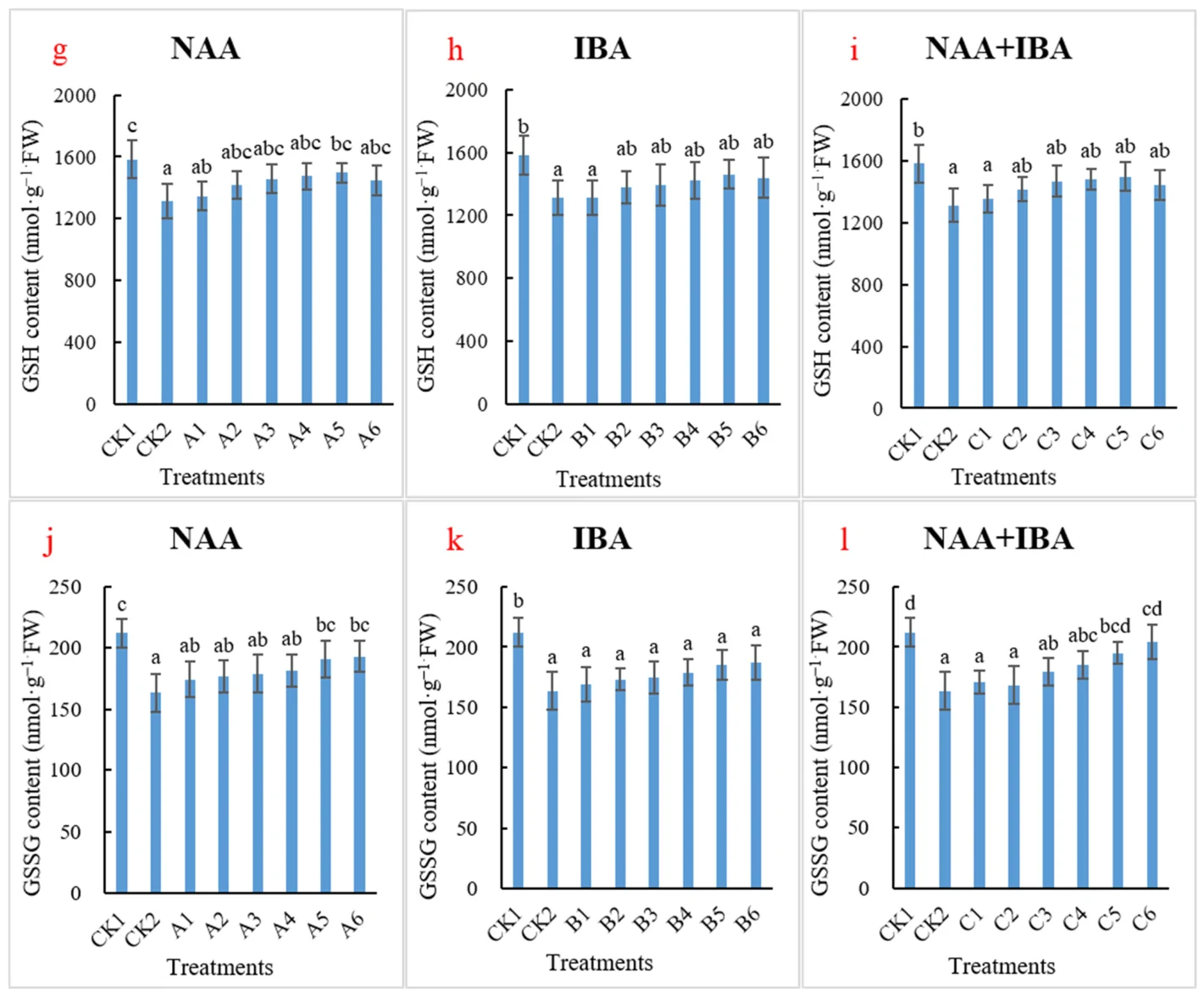
APX activities as well as AsA, GSH, and GSSG contents in rice seedlings derived from irradiated seeds soaked in auxin solutions. (**a**,**d**,**g**,**j**): Seeds soaked in NAA solution at concentrations of 0.02 (A1), 0.05 (A2), 0.1 (A3), 0.2 (A4), 0.4 (A5), and 0.8 (A6) mg/L. (**b**,**e**,**h**,**k**): Seeds soaked in IBA solution at concentrations of 0.06 (B1), 0.15 (B2), 0.25 (B3), 0.5 (B4), 1.0 (B5), and 2.0 (B6) mg/L. (**c**,**f**,**i**,**l**): Seeds soaked in NAA + IBA solution at combined concentrations of C1 (A1 + B1), C2 (A2 + B2), C3 (A3 + B3), C4 (A4 + B4), C5 (A5 + B5), and C6 (A6 + B6) mg/L. CK1: normal seeds; CK2: irradiated seeds without auxin treatment.

**Figure 5:**
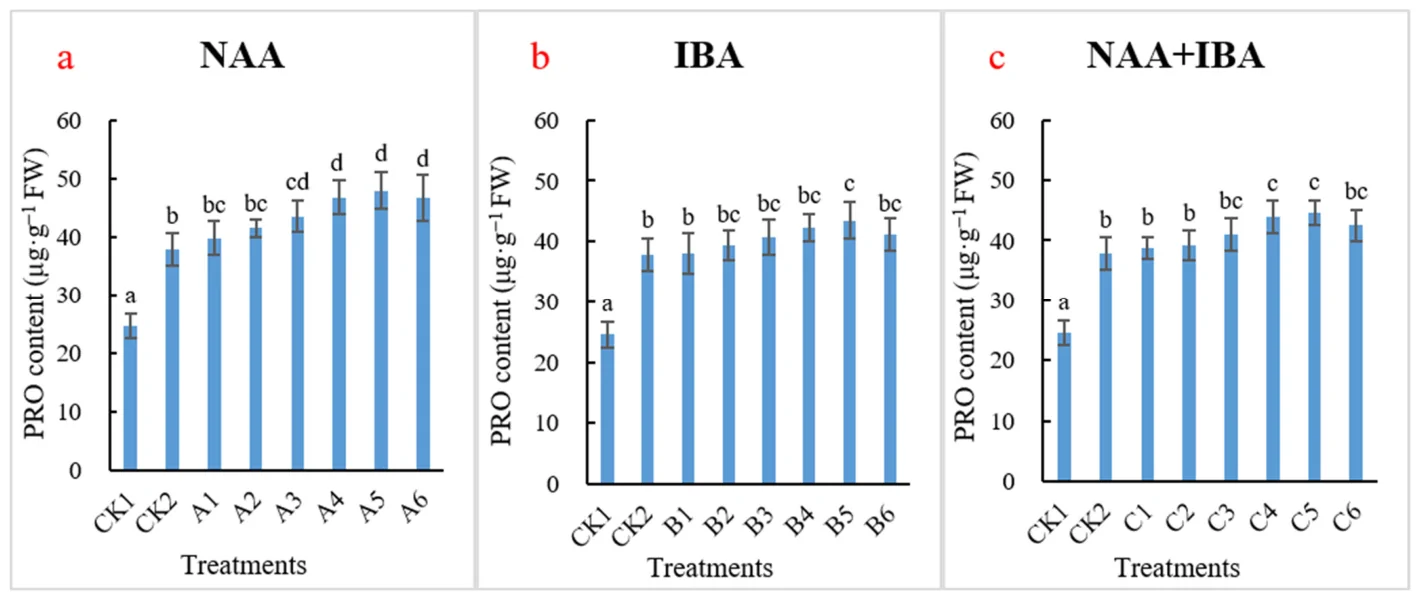
Proline contents in rice seedlings derived from irradiated seeds soaked in auxin solutions. (**a**): Seeds soaked in NAA solution at concentrations of 0.02 (A1), 0.05 (A2), 0.1 (A3), 0.2 (A4), 0.4 (A5), and 0.8 (A6) mg/L. (**b**): Seeds soaked in IBA solution at concentrations of 0.06 (B1), 0.15 (B2), 0.25 (B3), 0.5 (B4), 1.0 (B5), and 2.0 (B6) mg/L. (**c**): Seeds soaked in NAA + IBA solution at combined concentrations of C1 (A1 + B1), C2 (A2 + B2), C3 (A3 + B3), C4 (A4 + B4), C5 (A5 + B5), and C6 (A6 + B6) mg/L. CK1: normal seeds; CK2: irradiated seeds without auxin treatment.

**Figure 6:**
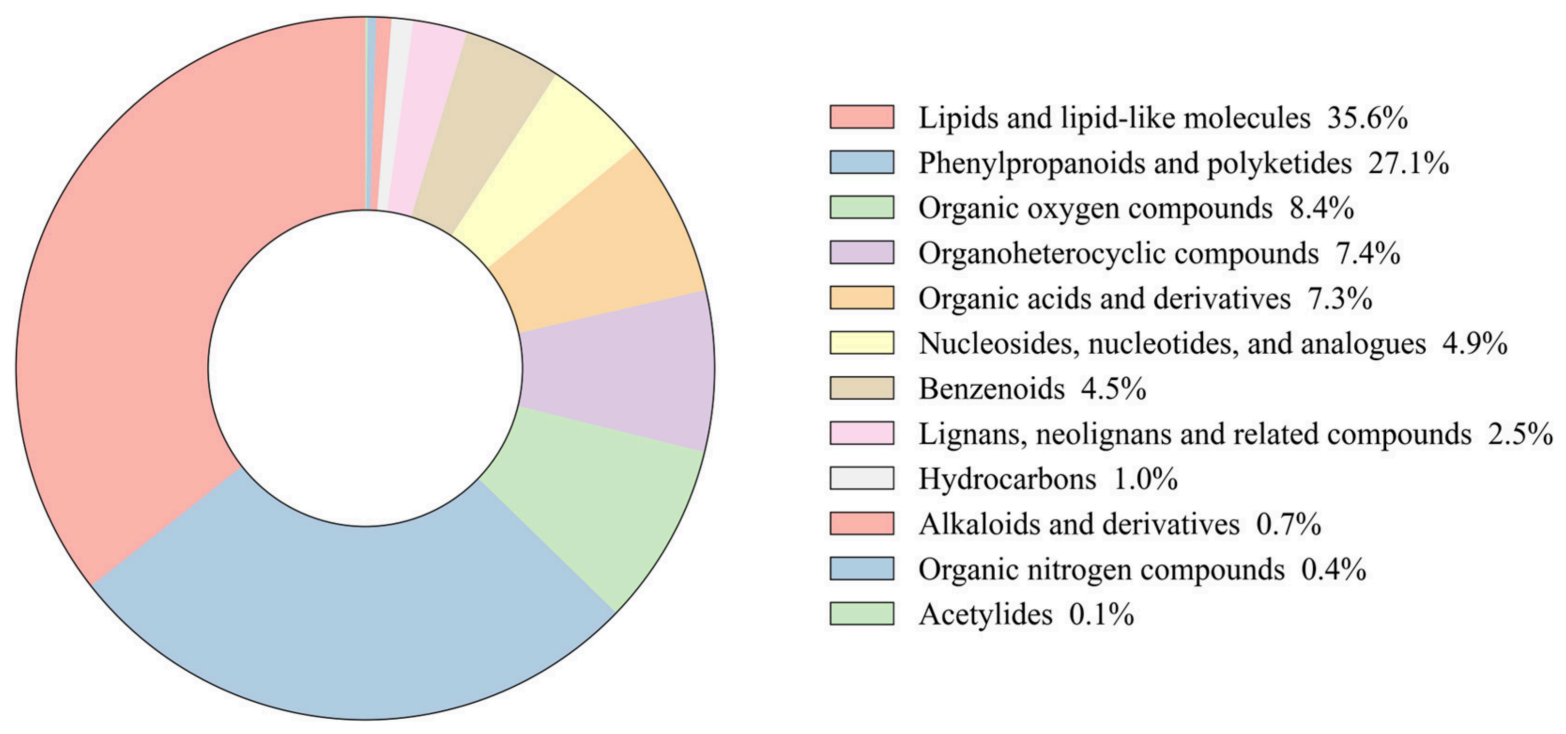
Classification of metabolites identified in rice seedling shoots in different comparison groups.

**Figure 7:**
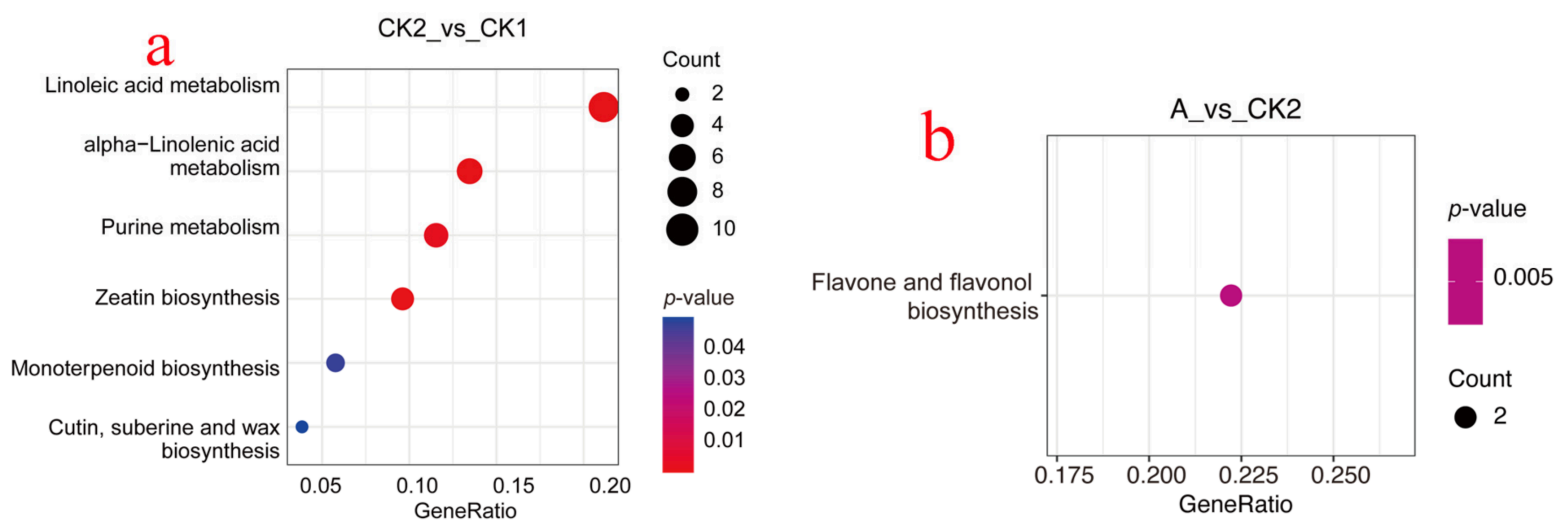

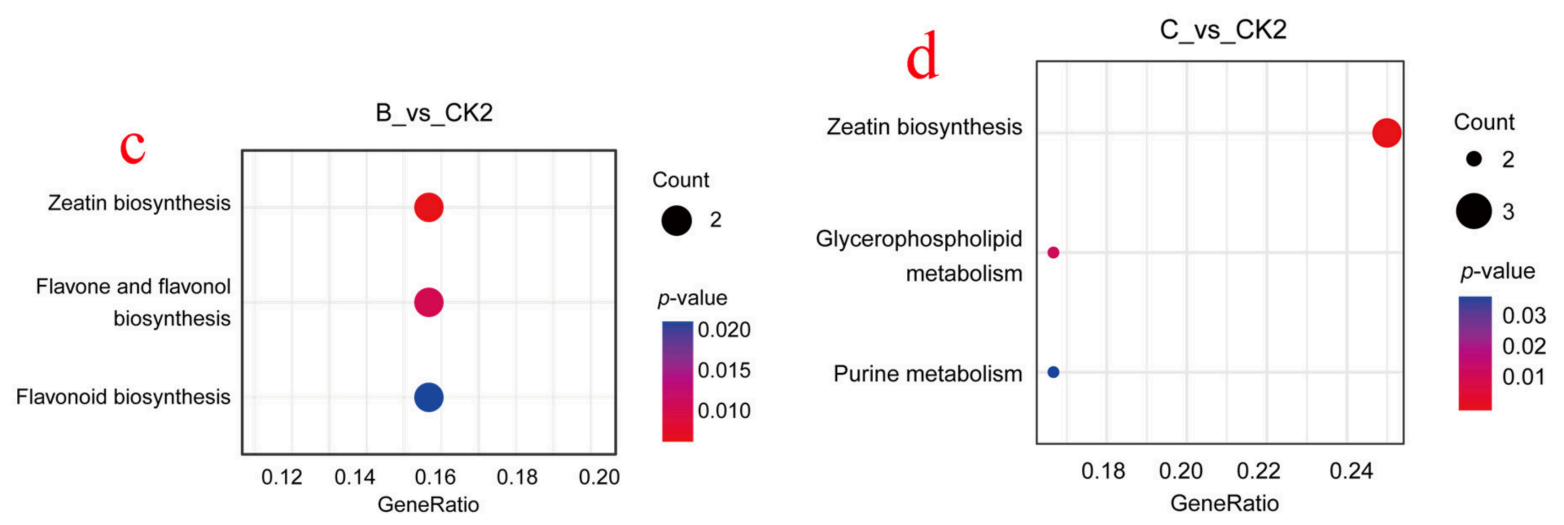
KEGG pathway enrichment analysis of DAMs among comparison groups. (**a**): Metabolic pathways enriched between CK2 and CK1. (**b**): pathways enriched between Group A and CK2. (**c**): Pathways enriched between Group B and CK2. (**d**): Pathways enriched between Group C and CK2.

**Figure 8:**
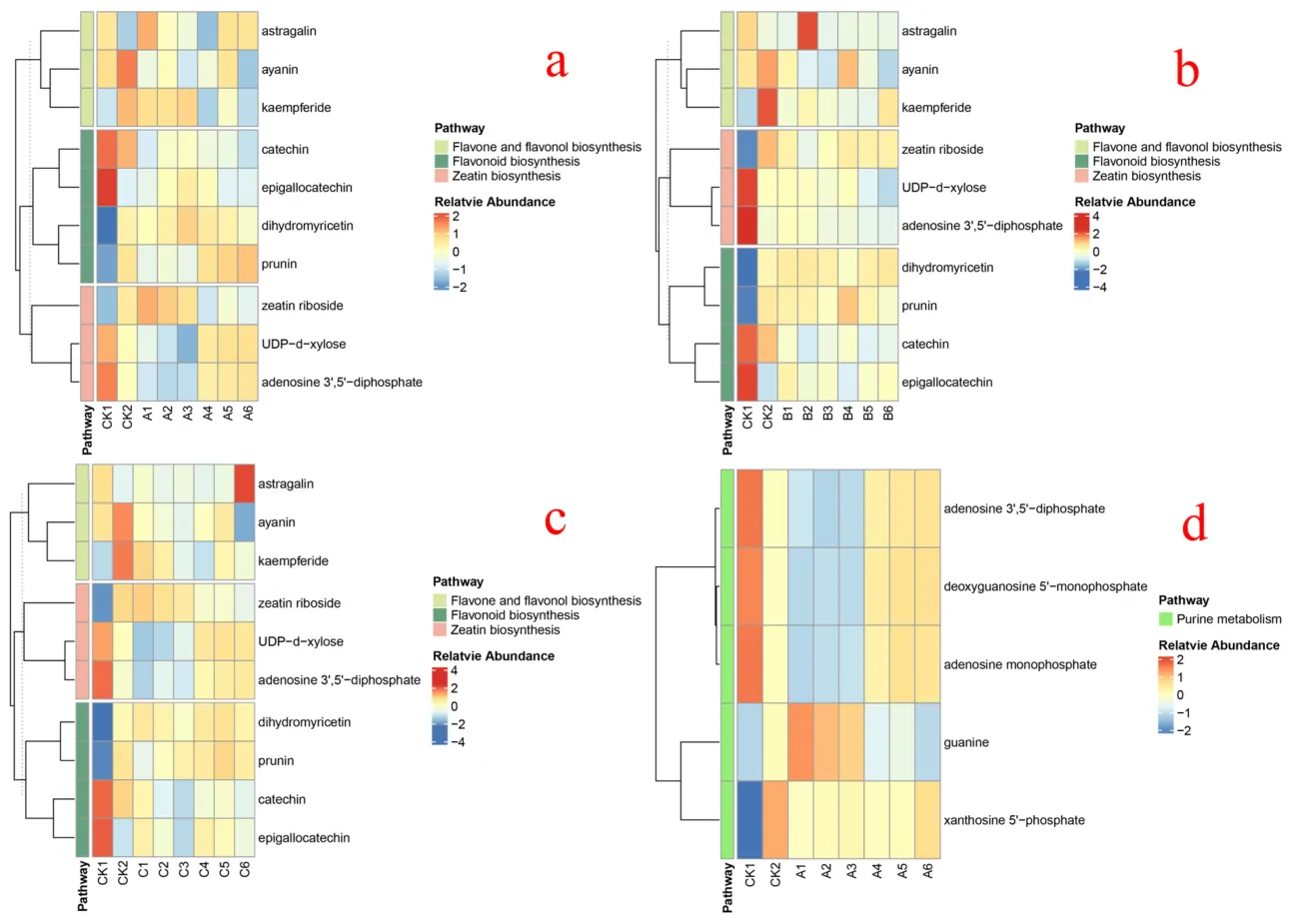

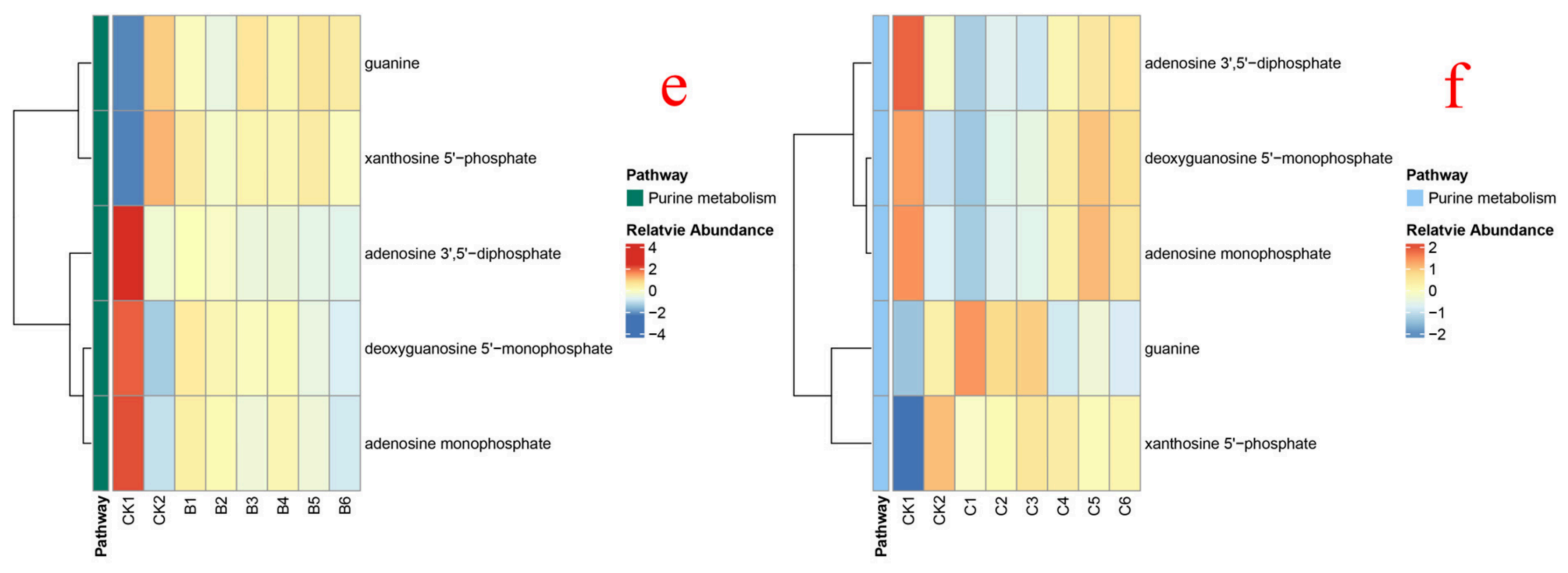
Heatmap of differentially accumulated metabolites in mutant seedlings derived from irradiated seeds treated with solutions containing NAA, IBA, and NAA + IBA at different concentrations. The color gradient (blue to red) represents the relative abundance of individual metabolites among samples. (**a**): Flavonoid and zeatin biosynthesis metabolites under NAA treatments. (**b**): Flavonoid and zeatin biosynthesis metabolites under IBA treatments. (**c**): Flavonoid and zeatin biosynthesis metabolites under combined NAA and IBA treatments. (**d**): Purine metabolism metabolites under NAA treatments. (**e**): Purine metabolism metabolites under IBA treatments. (**f**): Purine metabolism metabolites under combined NAA and IBA treatments.

**Figure 9:**
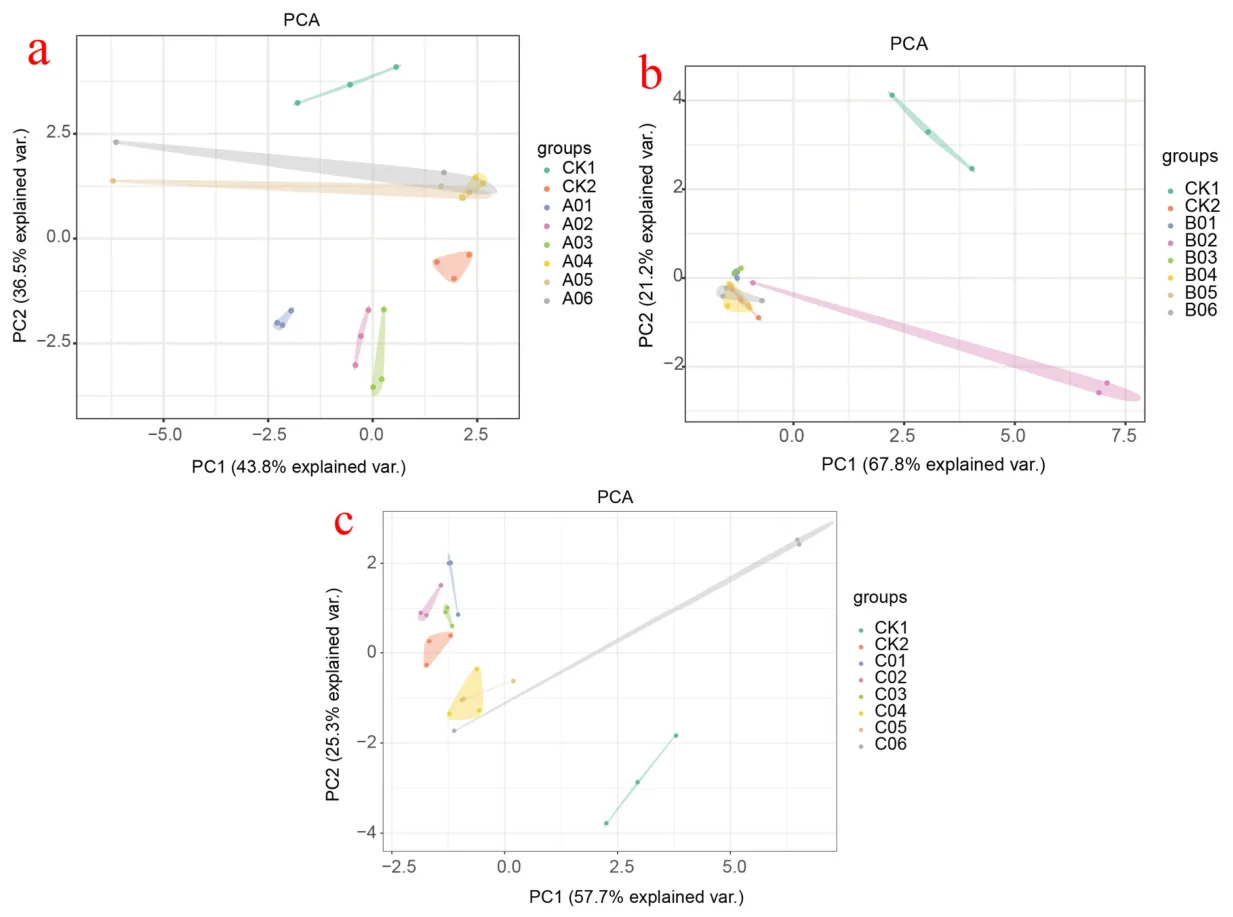
PCA of the metabolome of mutant seedlings derived from irradiated seeds treated with different auxin concentrations. (**a**): PCA plot for NAA treatments. (**b**): PCA plot for IBA treatments. (**c**): PCA plot for combined NAA and IBA treatments.

**Table 1:** Germination and seedling survival rates of irradiated rice seeds soaked in auxin solutions.

Treatments	CK1	CK2	A1	A2	A3	A4	A5	A6
Germination rate (%)	92.0 ± 3.0 ^a^	94.0 ± 2.6 ^a^	89.7 ± 2.5 ^a^	89.3 ± 2.5 ^a^	90.0 ± 2.6 ^a^	92.7 ± 3.1 ^a^	90.7 ± 3.8 ^a^	89.0 ± 1.0 ^a^
Seedling survival rate (%)	87.3 ± 2.5 ^a^	58.3 ± 4.2 ^c^	79.7 ± 4.2 ^b^	76.7 ± 4.0 ^b^	74.3 ± 5.5 ^b^	81.7 ± 3.8 ^ab^	79.7 ± 2.1 ^b^	80.0 ± 3.6 ^b^
Treatments	CK1	CK2	B1	B2	B3	B4	B5	B6
Germination rate (%)	92.0 ± 3.0 ^a^	94.0 ± 2.6 ^a^	93.7 ± 1.5 ^a^	93.0 ± 1.0 ^a^	89.3 ± 4.2 ^a^	89.7 ± 3.5 ^a^	92.0 ± 3.0 ^a^	89.7 ± 3.1 ^a^
Seedling survival rate (%)	87.3 ± 2.5 ^a^	58.3 ± 4.2 ^c^	77.3 ± 3.2 ^b^	73.7 ± 4.0 ^b^	79.7 ± 4.2 ^b^	78.7 ± 6.1 ^b^	75.3 ± 4.6 ^b^	79.0 ± 3.0 ^b^
Treatments	CK1	CK2	C1	C2	C3	C4	C5	C6
Germination rate (%)	92.0 ± 3.0 ^a^	94.0 ± 2.6 ^a^	91.3 ± 1.5 ^a^	91.0 ± 2.6 ^a^	91.7 ± 1.5 ^a^	95.3 ± 1.2 ^a^	92.7 ± 3.8 ^a^	90.7 ± 3.1 ^a^
Seedling survival rate (%)	87.3 ± 2.5 ^a^	58.3 ± 4.2 ^c^	83.3 ± 2.1 ^ab^	82.3 ± 2.3 ^ab^	80.3 ± 3.1 ^b^	86.7 ± 2.1 ^a^	81.7 ± 4.2 ^ab^	80.3 ± 3.8 ^b^

The lowercase letters in each column represent significance at the 0.05 level (Duncan’s multiple range test).

## Data Availability

The raw data supporting the conclusions of this article will be made available by the authors without undue reservation.

## Supplementary Materials

The following supporting information can be downloaded at: https://www.mdpi.com/article/10.3390/antiox15101359/s1. Figure S1: The plastic hydroponics pot for rice seedling culture CK1 without irradiated rice seedling growth state.
