# Supplementary material for: Exogenous NAA and IBA Alleviate the Adverse Effects of Gamma Radiation Stress and Increase Rice Seedling Survival Rates by Modulating Redox Balance and Metabolomic Regulation
Source: Antioxidants (Basel). 2026 Oct 20;15(10):1359. doi: 10.3390/antiox15101359 (PMC13701338; doi:10.3390/antiox15101359)
Supplement: Supplementary file 1 [file antioxidants-15-01359-s001.zip › antioxidants-4548100-supplementary.pdf]

Supplementary figure:

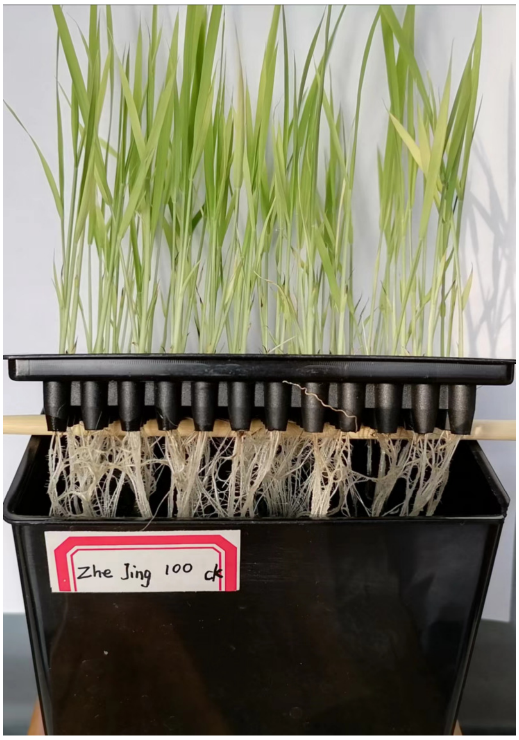

Figure S1: The plastic hydroponics pot for rice seedling culture.
CK1 without irradiated rice seedling growth state.
